# The causal relationship between antihypertensive drugs and depression: a Mendelian randomization study of drug targets

**DOI:** 10.3389/fendo.2024.1411343

**Published:** 2024-08-09

**Authors:** Zixian Yang, Jinshuai Li, Peichu Huang, Zhichang Li, Jianfeng He, Dongchun Cai, Yuzheng Lai

**Affiliations:** ^1^ Department of Neurology, Guangdong Provincial Hospital of Integrated Traditional Chinese and Western Medicine, Foshan, Guangdong, China; ^2^ The Fourth Clinical Medical College of Guangzhou University of Chinese Medicine, Shenzhen, Guangdong, China; ^3^ Nanhai District Hospital of Traditional Chinese Medicine of Foshan City, Foshan, Guangdong, China

**Keywords:** antihypertensive drugs, depression, Mendelian randomization, beta-blockers, Calcium Channel Blockers

## Abstract

**Background:**

Depression ranks as a leading contributor to the global disease burden. The potential causal relationship between the use of antihypertensive medications and depression has garnered significant interest. Despite extensive investigation, the nature of this relationship remains a subject of ongoing debate. Therefore, this study aims to evaluate the influence of antihypertensive medications on depression by conducting a Mendelian randomization study focused on drug targets.

**Method:**

We focused on the targets of five antihypertensive drug categories: ACE Inhibitors (ACEIs), Angiotensin II Receptor Antagonists (ARBs), Calcium Channel Blockers (CCBs), Beta-Blockers (BBs), and Thiazide Diuretics (TDs). We collected single-nucleotide polymorphisms (SNPs) associated with these drug targets from genome-wide association study (GWAS) statistics, using them as proxies for the drugs. Subsequently, we conducted a Mendelian randomization (MR) analysis targeting these drugs to explore their potential impact on depression.

**Results:**

Our findings revealed that genetic proxies for Beta-Blockers (BBs) were associated with an elevated risk of depression (OR [95%CI] = 1.027 [1.013, 1.040], p < 0.001). Similarly, genetic proxies for Calcium Channel Blockers (CCBs) were linked to an increased risk of depression (OR [95%CI] = 1.030 [1.009, 1.051], p = 0.006). No significant associations were identified between the genetic markers of other antihypertensive medications and depression risk.

**Conclusion:**

The study suggests that genetic proxies associated with Beta-Blockers (BBs) and Calcium Channel Blockers (CCBs) could potentially elevate the risk of depression among patients. These findings underscore the importance of considering genetic predispositions when prescribing these medications, offering a strategic approach to preventing depression in susceptible individuals.

## 1 Introduction

Depression, recognized as the most prevalent mental disorder globally (1), is projected by the World Health Organization to become the primary cause of disease burden worldwide by 2030 (2). This condition, due to its adverse impacts on both physical and mental well-being, has emerged as a major public health challenge and is currently the foremost cause of disability across the globe (3). Among patients with mental illnesses, there is a high prevalence of hypertension, and the diverse categories of antihypertensive medications, which act on various targets, can have differing impacts on depression. Widely utilized in the routine management of hypertension, antihypertensive medications including ACE Inhibitors (ACEIs), Angiotensin Receptor Blockers (ARBs), Calcium Channel Blockers (CCBs), Beta-Blockers (BBs), and Thiazide Diuretics (TDs) play a crucial role in controlling blood pressure (4) Grasping the potential of these antihypertensive drugs to cause, worsen, or alleviate neuropsychiatric symptoms is crucial for clinicians to make informed prescription decisions for patients with comorbid conditions.

At present, epidemiological evidence is inconclusive, as reported findings are contradictory. Observational studies have identified a differential association between various categories of antihypertensive drugs and the incidence of depression (5). A prior systematic review of double-blind randomized controlled trials revealed that Beta-Blockers (BBs) do not influence the risk of depression (6). Conversely, other research has proposed that BBs might possess antidepressant and anti-anxiety properties (7). Randomized clinical trials (RCTs) represent the gold standard for establishing the causal effects of drug treatments. Yet, RCTs are costly and can be subject to unavoidable confounding factors, potentially resulting in inconsistent outcomes. Drug target Mendelian Randomization (MR) analysis employs genetic variations that simulate the pharmacological inhibition of drug genetic targets as instrumental variables. This approach, via regression analysis, uncovers causal inferences regarding the potential impact of drug genetic targets on specific outcomes and enables the estimation of long-term medication effects on these outcomes (8, 9). Currently, Mendelian Randomization (MR) is being increasingly utilized to investigate the causal effects of antihypertensive drugs on diverse diseases (10, 11). The objective of this study is to employ drug target MR to examine the causal link between different categories of antihypertensive drugs and the risk of developing depression.

## 2 Methods

### 2.1 Obtaining GWAS summary-level statistics

This drug-target Mendelian Randomization (MR) study was carried out using publicly accessible summary-level statistics from Genome-Wide Association Studies (GWAS). A detailed summary of the GWAS summary-level statistics employed is provided in **Supplementary Table 1**. The summary-level statistics for systolic blood pressure (SBP) are derived from a recent large-scale GWAS meta-analysis (GWAS ID: ieu-b-38), encompassing data from over 750,000 Europeans across extensive cohort populations, including the UK Biobank and the International Blood Pressure Consortium (12). The GWAS summary statistics for depression were obtained from the FinnGen consortium database, R9 version (https://storage.googleapis.com/finngen-public-data-r9/summary_stats/finngen_R9_F5_DEPRESSIO.gz), encompassing 43,280 cases and 329,192 controls (13). The GWAS summary statistics for Coronary Artery Disease (CAD) originate from the CARDIoGRAMplusC4D consortium (GWAS ID: ebi-a-GCST005195), comprising 122,733 cases and 424,528 controls (14).

### 2.2 Selection of instrumental variables

**Figure 1:**
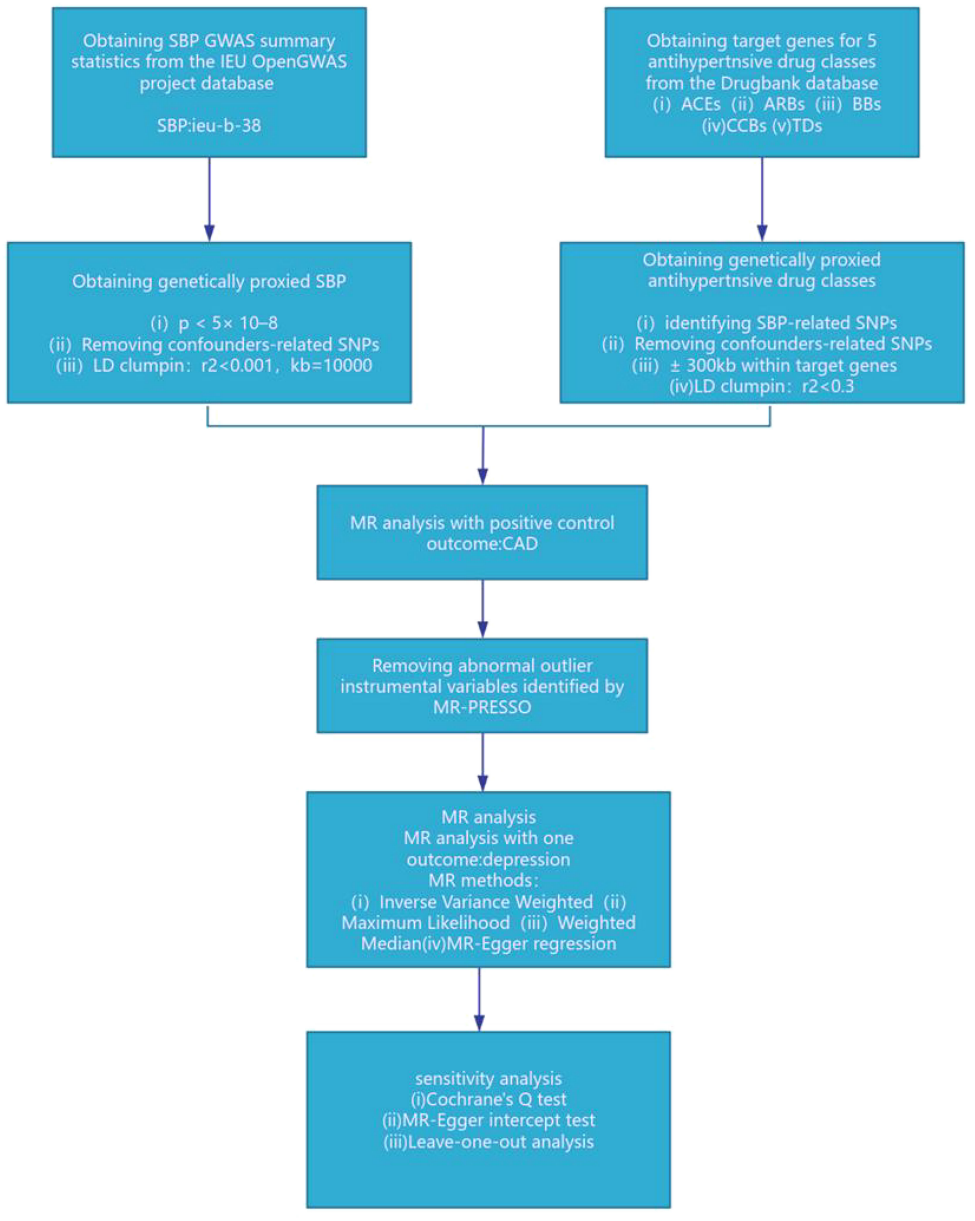
SBP, systolic blood pressure; ACEIs, ACE inhibitors; ARBs, angiotensin II receptor antagonists; BBs, beta-adrenergic antagonists; CCBs, calcium channel blockers; TDs, thiazide diuretics; CAD, coronary artery disease.

We employed genetic variants that simulate the effect of reducing systolic blood pressure (SBP) from specific antihypertensive drug target genes within ACEIs, ARBs, CCBs, BBs, and TDs, as delineated in published research (10, 11, 15–17). Specifically, we pinpointed 20 pharmacological target genes associated with blood pressure levels, as determined through the DrugBank database (10) (**Supplementary Material**: **Supplementary Table 1**). Instrumental variables were chosen for their genome-wide significance level linked to SBP (p < 5×10^–8), focusing on single nucleotide polymorphisms (SNPs) situated within ±300kb of the targeted drug genes (refer to **Figure 1**). To mitigate the impact of strong linkage disequilibrium (LD) on the findings, we established an LD threshold (r^2 < 0.3) (18). An F statistic exceeding 10 signifies a robust association between the SNP and the exposure, leading us to include only SNPs with an F statistic over 10. The F statistic was computed using the formula: F = Beta^2/SE^2 (19). The Mendelian Randomization (MR) assumption mandates that SNPs must not have a direct relationship with the outcome and cannot influence the outcome through any confounding factors aside from the exposure. Consequently, we utilized the PhenoScanner website (http://www.phenoscanner.medschl.cam.ac.uk/) to eliminate SNPs directly associated with the outcome and those linked to confounding factors of the outcome (20). Recognized risk factors for depression encompass psychological factors, behavioral lifestyle habits, age, medication adherence, among others (21). We retained 1 significant SNP for ACEIs, 22 significant SNPs for BBs, and 12 significant SNPs for CCBs. To confirm the validity of the genetic proxies for antihypertensive drugs, we further explored the relationship between these genetic proxies and Coronary Artery Disease (CAD), aligning our findings with prior meta-analyses of clinical trials (22, 23). Considering the established cardiovascular protective properties of antihypertensive medications, they were utilized as a positive control.

## 3 Data analysis

Initially, we aligned the exposure-related drug target instrumental variables with the outcome dataset, excluding SNPs that exhibit palindromic structures (24). Subsequent analyses employed methods such as Inverse Variance Weighted (IVW), Weighted Median, MR Egger, Simple Mode, and Weighted Mode. Among these, the IVW method is the most frequently utilized (25) and is deemed pivotal in Mendelian Randomization analysis. Therefore, should the outcomes of these methods diverge, the IVW results are prioritized as the primary findings. Heterogeneity tests utilized the MR Egger and IVW methods, employing Cochrane’s Q value to evaluate the heterogeneity among genetic instruments. A p-value greater than 0.05 signifies the absence of significant heterogeneity. When heterogeneity is detected, the Inverse Variance Weighted (IVW) random effects model is employed to minimize the impact of heterogeneity. Conversely, in the absence of heterogeneity, the IVW fixed effects model is utilized. The MR Egger regression equation and MR-PRESSO are utilized to evaluate the pleiotropy of genetic instruments, where a p-value greater than 0.05 indicates no significant pleiotropy (26). Furthermore, the MR-PRESSO test is capable of identifying outliers, and should outliers be detected, MR analysis is repeated after their exclusion. Furthermore, the MR-PRESSO test is capable of identifying outliers, and should outliers be detected, MR analysis is repeated after their exclusion. To guarantee that our findings are not disproportionately affected by any individual SNP, a leave-one-out analysis strategy is adopted, where each SNP is sequentially excluded and the outcomes are assessed using the IVW method. Data analysis was performed in R version 4.3.2, utilizing the MR-PRESSO and TwoSampleMR packages (26, 27).

## 4 Results

### 4.1 Identification of instrumental variables

**Figure 2:**
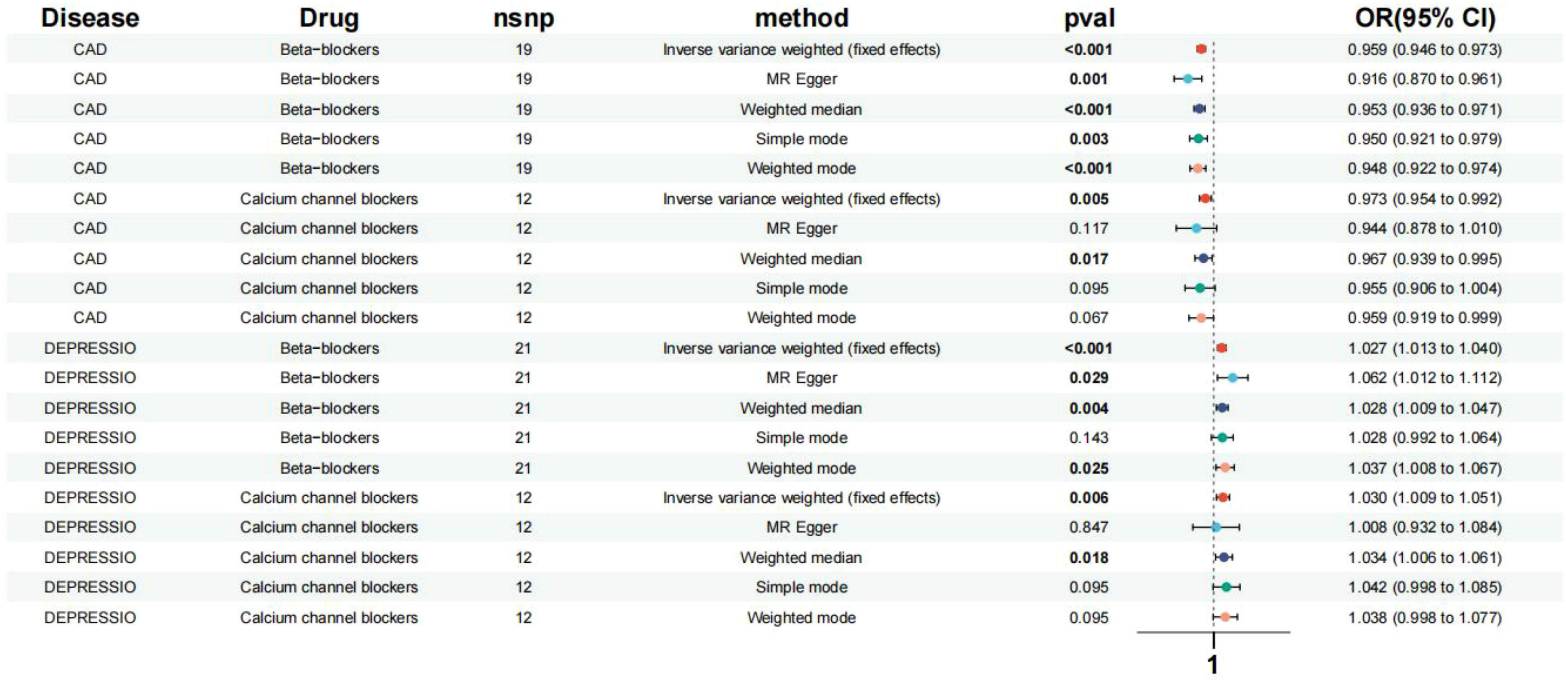
Effects of the β-blockers and the calcium channel blockers on depression, CAD. Based on exposure data from the FinnGen consortium database (version R9).

This study pinpointed SNPs associated with systolic blood pressure (SBP) influenced by various categories of antihypertensive drugs, revealing 1 SNP for ACEIs, 21 SNPs for BBs, and 12 SNPs for CCBs. Moreover, the values of SNPs mediating systolic blood pressure (SBP) through antihypertensive drugs were all greater than 10. Given that ACEIs had only a single SNP and considering the significance of the Inverse Variance Weighted (IVW) method for summarizing the effects of multiple loci in Mendelian Randomization (MR) analysis involving multiple SNPs, ACEIs were omitted from the subsequent MR analysis (**Supplementary Material**: **Supplementary Table 2**). The positive control outcomes for the genetic proxies of antihypertensive drugs in relation to Coronary Artery Disease (CAD) aligned with the results of clinical trials, affirming the reliability of these instrumental SNPs (**Figure 2**).

### 4.2 The causal relationship between antihypertensive drugs and depression

Based on the Inverse Variance Weighted (IVW) analysis, genetic proxies for Beta-Blockers (BBs) were found to elevate the risk of depression in patients (Odds Ratio [OR] [95% Confidence Interval [CI]] = 1.027 [1.013, 1.040], p < 0.001). Similarly, genetic proxies for Calcium Channel Blockers (CCBs) were associated with an increased risk of depression (OR [95%CI] = 1.030 [1.009, 1.051], p = 0.006) (**Figure 2**). No significant association was observed between the genes of other antihypertensive drugs and the risk of depression.

## 5 Sensitivity analysis

**Figure 3:**
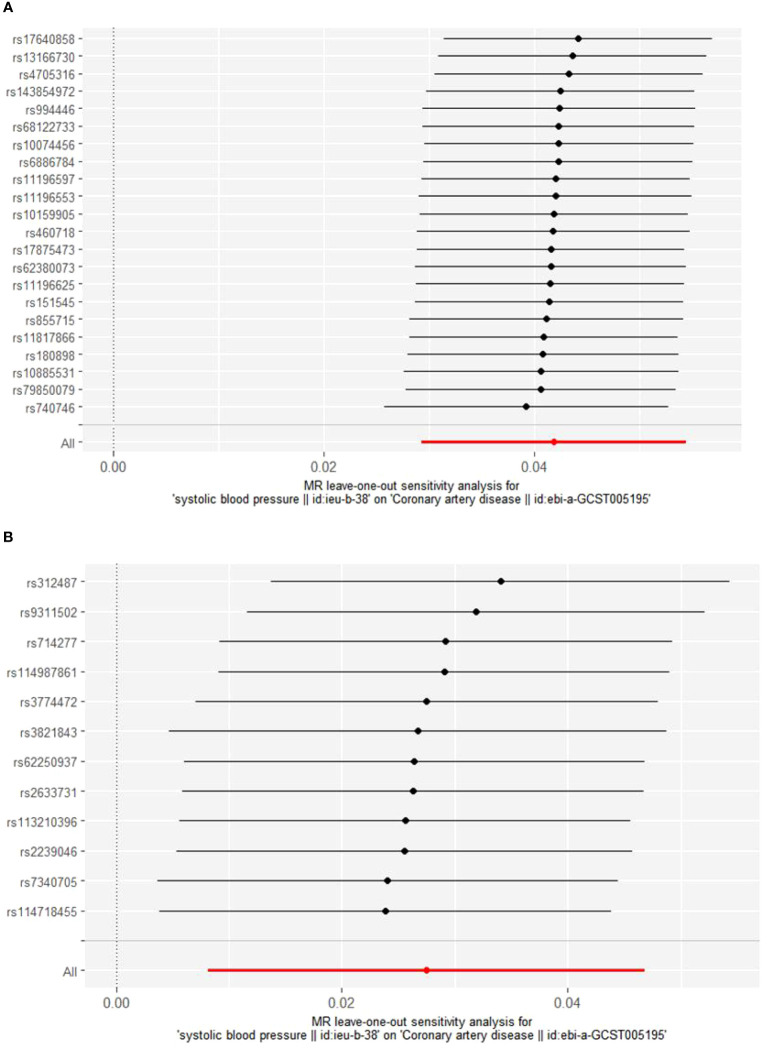
Sensitivity analysis of BBs **(A)** and CCBs **(B)** on CAD.

**Figure 4:**
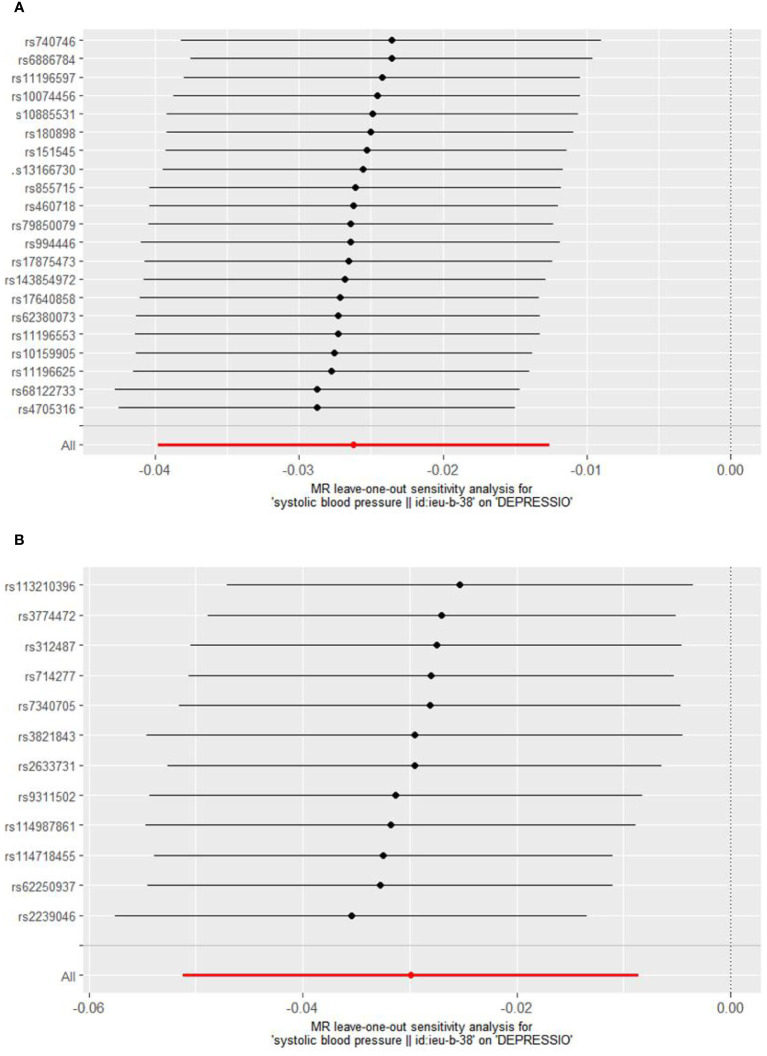
Sensitivity analysis of BBs **(A)** and CCBs **(B)** on Depression.

Cochrane’s Q test was employed to evaluate heterogeneity, revealing no significant heterogeneity (**Supplementary Material**: **Supplementary Table 3**). Consequently, the Inverse Variance Weighted Fixed Effects (IVWFE) model was utilized for Mendelian Randomization (MR) analysis. The MR-Egger regression and MR-PRESSO global test methods were applied to evaluate pleiotropy, with neither method suggesting the existence of pleiotropy (**Supplementary Material**: **Supplementary Table 3**). Additionally, the leave-one-out analysis confirmed the robustness of our findings (**Figures 3**, **4**).

## 6 Discussion

In this extensive Mendelian Randomization (MR) analysis, which encompassed 43,280 cases and 329,192 controls, we explored the influence of various categories of antihypertensive drug targets on depression risk. Our findings suggest that, genetically, Beta-Blockers (BBs) and Calcium Channel Blockers (CCBs) may elevate the risk of depression in patients.

Observational studies have explored the effects of antihypertensive drugs on depression risk, yet the findings have been varied and inconclusive (4–6). ACE Inhibitors (ACEIs), which target the renin-angiotensin system (RAS), are thought to offer protective benefits for cognition, depression, and anxiety. Similarly, Beta-Blockers (BBs) are believed to have antidepressant and anti-anxiety effects (7). The findings appear to indicate that antihypertensive drugs may have the potential to prevent depression. However, an observational study utilizing linked medical data in Scotland reported that no antihypertensive medication was effective in preventing the onset of new depression cases (28). A genomic study further revealed that single nucleotide polymorphisms (SNPs) in CACNA1C, identified as a cross-disorder risk gene, are linked to the prognosis of psychiatric disorders (29, 30), echoing the results of our investigation. A connection between inflammation and psychiatric disorders has been identified in the context of Calcium Channel Blockers (CCBs), which are suggested to elevate TNF-alpha (an inflammatory factor) (31). Furthermore, inflammation is implicated in the development of psychiatric disorders (32). In recent years, there has been a growing identification of hypertensive patients who either have psychiatric disorders or are highly susceptible to them. Despite this, considerable debate persists regarding the depression risk associated with antihypertensive drugs, compounded by a scarcity of related research. This underscores the critical need to elucidate the relationship between these medications and depression. Observational studies are often influenced by numerous unavoidable confounding factors, resulting in inconsistent outcomes. Recently, Mendelian Randomization (MR) research has gained popularity for its ability to elucidate the relationship between exposure and outcome through a genetic lens, significantly reducing the influence of confounding factors on the findings (33).

Our study presents several advantages. Primarily, it is the inaugural Mendelian Randomization (MR) investigation focusing on the target genes of various categories of antihypertensive drugs in relation to depression. Secondly, our study effectively reduced the impact of confounding factors, with sensitivity analysis further confirming the robustness of our findings. Lastly, the genetic proxies for Beta-Blockers (BBs) and Calcium Channel Blockers (CCBs) were identified as risk factors for depression, potentially offering new directions for future drug development. However, our study also encounters certain limitations. Although using a sample of European descent effectively reduces bias from population stratification, caution is warranted when extrapolating findings to other populations due to potential inter-ethnic genetic variations. Unfortunately, due to insufficient data, we cannot explore the effects of blood pressure and antihypertensive drugs on other ethnic groups. Second, our analysis reflects only the long-term effects of lifelong inhibition of drug targets. Consequently, the relationship between short-term medication use and disease risk remains unexplored. In addition, using GWAS summary data and SNPs as proxies for drug effects has limitations, including the inability to accurately reflect the complex actions of drugs, estimation errors, limited external validity, and potential confounding factors. These constraints impact the reliability and generalizability of research findings. To address these issues, future studies should integrate diverse data sources and methodologies, and enhance statistical models to improve the precision and practical application of results. Furthermore, given the importance of personalized medicine, there is a notable gap between genetic findings and clinical practice. To bridge this gap, it is recommended to incorporate the patient’s overall health status into the formulation of specific clinical guidelines and to validate the practical application of genetic information through large-scale clinical trials. While clinicians can use a patient’s genetic information as a reference for prescribing antihypertensive drugs, decisions should not rely solely on this information. Comprehensive evaluation of factors such as the patient’s overall health status, concurrent medication use, and lifestyle is essential. Using genetic information for drug prescriptions raises ethical issues, including genetic discrimination, privacy protection, and informed consent. Measures should be taken to prevent genetic discrimination, safeguard data privacy, ensure informed consent from patients, and promote fairness in medical practices. Future research efforts should focus on establishing robust legal protections and exploring ethical frameworks tailored to the application of genetic information across diverse cultural contexts. Lastly, our study focuses solely on the causal effect concerning the risk of disease onset. The impact of long-term medication use on disease prognosis remains to be elucidated.

## 7 Conclusions

Genetic proxies for Beta-Blockers (BBs) and Calcium Channel Blockers (CCBs) have been identified as increasing the risk of depression in patients. However, comprehensive basic research and randomized controlled trials are essential in the future to elucidate their underlying mechanisms. To date, no evidence has been found linking genes of other antihypertensive drugs to the risk of depression.

## Data Availability

Publicly available datasets were analyzed in this study. The summary statistics for the systolic blood pressure GWAS can be accessed at https://gwas.mrcieu.ac.uk/files/ieu-b-38/ieu-b-38.vcf.gz, and the summary statistics for depression can be accessed at https://storage.googleapis.com/finngen-public-data-r9/summary_stats/finngen_R9_F5_DEPRESSIO.gz.
