# Supplementary material for: The causal relationship between antihypertensive drugs and depression: a Mendelian randomization study of drug targets
Source: Front Endocrinol (Lausanne). 2024 Aug 9;15:1411343. doi: 10.3389/fendo.2024.1411343 (PMC11344258; doi:10.3389/fendo.2024.1411343)
Supplement: Supplementary file 1 [file DataSheet_1.pdf]

Table 1. Regions of target genes identified by the NCBI Gene database

| Drug class | Target gene | Chromosome | Position (GRCh37/hg19) |
|---|---|---|---|
| ACE inhibitor | ACE | 17 | 61554422-61575734 |
| Angiotensin II receptor antagonist | AGTR1 | 3 | 148415690-148460790 |
| | AGTR2 | X | 115301997-115306227 |
| Beta-blockers | ADRB1 | 10 | 115803625-115806663 |
| | ADRB2 | 5 | 148206174-148208186 |
| Calcium channel blocker | CACNA1A | 19 | 13317256-13617293 |
| | CACNA1C | 12 | 2162153-2807116 |
| | CACNA1D | 3 | 53528638-53847760 |
| | CACNA1H | 16 | 1203106-1271768 |
| | CACNA1S | 1 | 201008640-201081554 |
| | CACNA2D1 | 7 | 81575760-82073272 |
| | CACNA2D2 | 3 | 50400044-50541675 |
| | CACNB1 | 17 | 37329706-37353922 |
| | CACNB2 | 10 | 18429353-18832486 |
| | CACNB3 | 12 | 49208263-49222724 |
| | CACNB4 | 2 | 152689285-152955681 |
| | CACNG1 | 17 | 65040670-65052913 |
| Thiazide diuretics | SLC12A1 | 15 | 48498499-48596275 |
| | SLC12A2 | 5 | 127419458-127525369 |
| | SLC12A3 | 16 | 56899119-56949762 |

| Table 2 Single-nucleotide polymorphisms (SNPs) related to SBP mediated by target genes of BBs, CCBs at genome-wide significance | | | | | | | | |
|---|---|---|---|---|---|---|---|---|
| Characteristics | chr | position | Beta | SE | P-value | SNP | effect_allele | other_allele |
| SBP mediated by BBs target genes | 5 | 148348395 | -0.3466 | 0.0374 | 1.87413E-20 | rs994446 | A | G |
| | 5 | 148410506 | -0.4618 | 0.0698 | 3.69318E-11 | rs10074456 | G | C |
| | 5 | 148344227 | -0.3102 | 0.0438 | 1.49417E-12 | rs6886784 | A | G |
| | 5 | 148471391 | -0.2552 | 0.0338 | 4.4269E-14 | rs62380073 | G | A |
| | 5 | 148346260 | 0.2014 | 0.0321 | 3.29503E-10 | rs17640858 | A | C |
| | 5 | 148498799 | -0.2229 | 0.0355 | 3.22701E-10 | rs4705316 | A | G |
| | 5 | 148373306 | 0.2435 | 0.0423 | 8.32703E-09 | rs13166730 | T | C |
| | 10 | 115700005 | -0.2932 | 0.0477 | 8.13205E-10 | rs151545 | A | C |
| | 10 | 115792787 | 0.4557 | 0.0342 | 1.42102E-40 | rs740746 | A | G |
| | 10 | 115814392 | 0.2999 | 0.0302 | 3.6266E-23 | rs10885531 | T | C |
| | 10 | 115823524 | -0.4394 | 0.0482 | 7.87771E-20 | rs855715 | T | G |
| | 10 | 115843445 | 0.4306 | 0.0664 | 8.89406E-11 | rs143854972 | A | G |
| | 10 | 115710997 | 0.6252 | 0.074 | 2.89401E-17 | rs11196553 | T | C |
| | 10 | 115721364 | 0.2764 | 0.0324 | 1.358E-17 | rs460718 | G | A |
| | 10 | 115790006 | -0.5804 | 0.0905 | 1.448E-10 | rs79850079 | A | G |
| | 10 | 115800294 | 0.3283 | 0.0552 | 2.65797E-09 | rs17875473 | T | C |
| | 10 | 115831533 | -0.3343 | 0.0406 | 1.68811E-16 | rs68122733 | G | A |
| | 10 | 115765397 | -0.5177 | 0.065 | 1.64702E-15 | rs180898 | C | A |
| | 10 | 115788094 | 0.2858 | 0.0458 | 4.22698E-10 | rs11196597 | A | G |
| | 10 | 115843990 | 0.2274 | 0.0395 | 8.28591E-09 | rs11196625 | A | G |
| | 10 | 115977065 | 0.3217 | 0.0563 | 1.102E-08 | rs10159905 | C | G |
| SBP mediated by CCBs target genes | 3 | 53612327 | -0.4338 | 0.077 | 1.75999E-08 | rs113210396 | T | G |
| | 3 | 53558012 | 0.3373 | 0.0335 | 6.55843E-24 | rs3821843 | A | G |
| | 3 | 53560321 | 0.2463 | 0.0355 | 3.87258E-12 | rs9311502 | C | T |
| | 3 | 53870318 | -0.3238 | 0.056 | 7.27897E-09 | rs62250937 | C | T |
| | 3 | 53464055 | 0.5102 | 0.0905 | 1.71799E-08 | rs114718455 | G | A |
| | 3 | 53605712 | 0.5289 | 0.0958 | 3.36001E-08 | rs114987861 | A | G |
| | 3 | 53738424 | 0.1963 | 0.0309 | 2.20699E-10 | rs2633731 | C | T |
| | 3 | 53545622 | -0.2194 | 0.0307 | 9.64717E-13 | rs312487 | C | T |
| | 3 | 53734443 | 0.2425 | 0.0322 | 4.87304E-14 | rs7340705 | C | T |
| | 3 | 53638200 | 0.1747 | 0.0303 | 8.21107E-09 | rs3774472 | G | A |
| | 12 | 2434419 | 0.2082 | 0.0322 | 9.57635E-11 | rs2239046 | A | G |
| | 12 | 2514270 | 0.1986 | 0.0333 | 2.37799E-09 | rs714277 | T | C |

**Table 3 The result of heterogeneity test and horizontal pleiotropic test for repeated analysis.**

| Drug | Outcome | Test | Method | P |
|---|---|---|---|---|
| BBs | CAD | Heterogeneity | Cochran's Q test | 0.942(QMR Egger) |
| BBs | CAD | Heterogeneity | Cochran's Q test | 0.907(QIVW) |
| BBs | CAD | Pleiotropy | MR-Egger regression | 0.185 |
| BBs | CAD | Pleiotropy | MR-PRESSO global test | 0.915 |
| CCBs | CAD | Heterogeneity | Cochran's Q test | 0.471(QMR Egger) |
| CCBs | CAD | Heterogeneity | Cochran's Q test | 0.483(QIVW) |
| CCBs | CAD | Pleiotropy | MR-Egger regression | 0.369 |
| CCBs | CAD | Pleiotropy | MR-PRESSO global test | 0.502 |
| BBs | Depression | Heterogeneity | Cochran's Q test | 0.523(QMR Egger) |
| BBs | Depression | Heterogeneity | Cochran's Q test | 0.462(QIVW) |
| BBs | Depression | Pleiotropy | MR-Egger regression | 0.180 |
| BBs | Depression | Pleiotropy | MR-PRESSO global test | 0.475 |
| CCBs | Depression | Heterogeneity | Cochran's Q test | 0.378(QMR Egger) |
| CCBs | Depression | Heterogeneity | Cochran's Q test | 0.433(QIVW) |
| CCBs | Depression | Pleiotropy | MR-Egger regression | 0.563 |
| CCBs | Depression | Pleiotropy | MR-PRESSO global test | 0.484 |
